# Stress, anxiety, and depression among healthcare workers during the COVID-19 pandemic in Kosovo

**DOI:** 10.1093/eurpub/ckac131.494

**Published:** 2022-10-25

**Authors:** F Arenliu Qosaj, S Merrill Weine, P Sejdiu, F Hasani, S Statovci, V Behluli, A Arenliu

**Affiliations:** 1 Master Program in Healthcare Management, College AAB, Prishtina, Kosovo; 2 Center for Global Health, University of Illinois, Chicago, USA; 3 Professional Services Department, Kosovo Medical Chamber, Prishtina, Kosovo; 4 Orthopedic Clinic, Kosovo Hospital University Clinical Services, Prishtina, Kosovo; 5 Nursing Department, Alma Mater Europaea, Campus College Rezonanca, Prishtina, Kosovo; 6 Professional Services Department, Kosovo Nursing Chamber, Prishtina, Kosovo; 7 Nursing Division, Ministry of Health, Government of the Republic of Kosovo, Prishtina, Kosovo; 8 Psychiatry Clinic, Kosovo Hospital University Clinical Services, Prishtina, Kosovo; 9 Professional Services Department, Kosovo Association of Psychologists, Prishtina, Kosovo; 10 Department of Psychology, Prishtina University, Prishtina, Kosovo

## Abstract

**Background:**

The COVID-19 pandemic has created a very large workload burden on health systems worldwide, Kosovo is no exception to this trend. A pandemic may have a negative impact on health care workers’ (HCWs) mental health. In this cross-sectional study, we assessed the self-reported prevalence of stress, anxiety, and depression and identified their predictive factors among HCWs in Kosovo.

**Methods:**

Data were collected on sociodemographics (sex, age, occupation, education, workplace) and the presence and severity of depression, anxiety, and stress through the 21-item Depression, Anxiety, and Stress Scale (DASS-21) online questionnaire. Descriptive statistics, t-tests, and linear logistic regression were used to analyze the data.

**Results:**

Of the 545 respondents, the majority were male (53.0%), under 60 years of age (94.7%), and married (81.7%). Most of them were physicians (78.2%), while the remaining respondents were nurses, midwives, and other health professionals (22%). The prevalence rates for moderate to extremely high levels of stress, anxiety, and depressive symptoms were 21.9%, 13.0%, and 13.9%, respectively. Nurses reported significantly higher mean scores for depression and anxiety than physicians (P < 0.05). Being married, having poor health, not exercising, and reporting ‘burnout’ from work significantly predicted higher levels of depressive, anxiety, and stress symptoms among health workers (P < 0.05).

**Conclusions:**

HCWs require specific national mental health intervention programs that will, among other effects, help raise awareness of the early recognition of symptoms related to stress, anxiety, depression, and burnout due to workload as well as the importance of regular physical exercise. These programs should be part of the national emergency preparedness, emergency response, and health sector strategy, aiming to build and sustain a resilient health system.

**Key messages:**

• During COVID-19 pandemic, certain factors predicted higher levels of mental health burden among HCWs.

• Addressing these factors require policy recommendations with concrete systemic interventions.

